# Education and Career Advancement Opportunities in Polish and English Nursing—A Comparative Study of Intensive Care Unit Nurses

**DOI:** 10.3390/nursrep14030128

**Published:** 2024-07-17

**Authors:** Ewelina Wasielewska, Piotr Kordel, Marcin Moskalewicz

**Affiliations:** 1 Department of Nursing Practices, Poznan University of Medical Sciences, Rokietnicka 2a, 60-806 Poznań, Poland; ewasielewska@ump.edu.pl; 2 Department of Anesthesiology and Intensive Care, Independent Public Healthcare Center of the Ministry of Interior and Administration Prof. Ludwik Bierkowski, Dojazd 34, 60-631 Poznań, Poland; 3 Philosophy of Mental Health Unit, Department of Social Sciences and the Humanities, Poznan University of Medical Sciences, Rokietnicka 7, 60-806 Poznań, Poland; kordel@ump.edu.pl; 4 Psychiatric Clinic, Heidelberg University, Voßstraße 4, 69115 Heidelberg, Germany; 5 Institute of Philosophy, Marie Curie-Sklodowska University, Marii Curie-Skłodowskiej 4, 20-031 Lublin, Poland; 6 IDEAS NCBR, Chmielna 69, 00-801 Warszawa, Poland

**Keywords:** nursing education, critical care nursing, continuing education, emigration and immigration, nursing staff, professional development

## Abstract

The aim of this study was to comparatively investigate education, job, and professional development satisfaction among intensive care unit nurses in Poland and England. A total of 258 ICU nurses from both countries were interviewed (72 Polish nurses working in Poland and 186 of various national backgrounds in England, including 50 of Polish origin). We used an 11-item structured survey followed by an open-ended qualitative interview retrospectively coded for statistical analysis. Regardless of national origin, nurses in England report significantly higher education satisfaction, attributed to better theoretical knowledge gain but not to other dimensions (such as practical knowledge or personal satisfaction). They also express greater satisfaction with job conditions regarding professional development, a state-of-the-art work environment, teamwork, and finance. The UK system is also considered significantly superior in promotion opportunities and participation in post-graduation training. In conclusion, systemic factors play a crucial role in career satisfaction and advancement in nursing, with the British band system having a clear advantage over the Polish one.

## 1. Introduction

### 1.1. Burnout in Nursing

Burnout is one of the leading reasons for leaving the nursing profession [1,2]. Low or inadequate nurse staffing levels, ≥12 h shifts, low schedule flexibility, time pressure, high job and psychological demands, low task variety and autonomy, negative nurse–physician relationships, poor supervisor and leader support, and negative team relationships combined with work–family conflicts contribute to high levels of burnout and compassion fatigue in nurses [3,4,5,6]. The type of working environment also contributes to nurses’ burnout, with nurses working at ICUs experiencing a higher level of burnout than the ones working at surgical wards [7,8]. Oncological nurses, apart from burnout, experience compassion fatigue [9].

### 1.2. Career Advancement in Nursing

All the factors mentioned above not only lead to burnout but also prevent nurses from seeking new professional opportunities [3,4,5,6]. Another issue hindering nurses’ professional growth is the limited number of possible career paths, typically directed toward leadership, academic, or educational work [10]. To attract more people to join the medical workforce and enhance nurse retention in the healthcare systems, healthcare systems regulators and organizations must provide nurses with clear career planning and development schemes [10,11]. In some countries, this is carried out better than in others, making them more attractive places to work as nurses; in addition, some countries offer higher earnings. Thus, career advancement in nursing is complicated and can be influenced by many systemic, professional, as well as personal challenges.

### 1.3. Nursing Migration to the UK from Poland

Joining the EU in 2004 enabled Polish workers to seek employment opportunities in Western European countries. Higher earnings and better working conditions were two of the most important reasons supporting the decision to emigrate from the home country. In the first year of Poland’s EU membership, one million Poles went to work abroad. In 2020, this number reached 2.2 million. Most Polish migrants chose to go to Germany, Great Britain, the Netherlands, Norway, and Italy [12]. This trend also applies to Polish nurses leaving Poland to work in other European countries. A study from 2016 showed that 12.4% of Polish nurses have already worked abroad, and almost 30% considered working abroad, especially in Germany and Great Britain [13]. This may cause a severe threat to the Polish healthcare system, as Poland already has one of the lowest numbers of nurses (5.7) per 1000 inhabitants among the 27 EU countries [14].

England is one of the most popular destinations for Polish nurses; this is the case for nurses from other countries as well. According to 2022 data, 76% of nurses and health visitors in England’s hospital and community health services reported being British nationals. The rest come from Asia (14%), mainly India and the Philippines, and the European Economic Area (5%), mainly from Ireland, Romania, Portugal, Spain, Italy, and Poland [15,16].

### 1.4. Aims of the Study

This study explored how intensive care nursing differs in Poland and England in terms of education, professional training and development, and job satisfaction. We hypothesized higher levels of education and job satisfaction, higher participation in post-graduate training, and higher levels of desire for career advancement (as exemplified by, for example, applying for promotion) among nurses working in England. We also assumed that these differences would reappear regardless of nationality when comparing Polish nurses working in Poland and England, enabling these differences to be attributed to systemic factors and not to the national background.

## 2. Materials and Methods

### 2.1. Sample

The studied sample consisted of 258 nurses working in intensive care units both in Poland (Wielospecjalistyczny Szpital im. J. Strusia in Poznan and Szpital im. J. B. Kraszewskiego in Puszczykowo, both having general-profile ICUs with 24 and 12 beds, respectively) and England (Royal Surrey County Hospital in Guilford with 35 beds, a general-profile ICU, and Papworth Hospital in Papworth Everard with 65 beds, a cardiological-profile ICU). All Polish nurses working in Poland were either already specialized in intensive care and anaesthesiology nursing or were in such training.

The UK hospitals covered by the study followed staff education and training standards approved by the NHS and NMC. Each new employee, regardless of whether they were from outside the UK or from within, was subjected to a membership program, which involved teaching and introducing a new person to work in a given department. Each person had a supervisor who introduced and assessed activities necessary to work in the ICU ward. After the program’s completion, an internal examination was held. The program lasted from 6 months to a year with the possibility of extension. There is no such program in Poland, where newly employed people are often taught by their peers on a shift-to-shift basis.

The study participants were recruited and directly interviewed by one of the authors [EW], who worked as a nurse in all the abovementioned units between 2018 and 2022. The interviews, framed as peers’ conversations, occurred shortly before or after a shared shift/duty. All the participants were informed that the collected data would be used for scientific research as the interviewer wrote down their answers.

A total of 28% (*n* = 72) of the sample consisted of nurses working in Poland and 72% (*n* = 186) consisted of British, Polish, Portuguese, Spanish, and Italian nurses working in England. It is worth noticing that the study started before Brexit; hence, the nationalities of internationally trained nurses working in England may differ from those in post-Brexit circumstances. As Table 1 illustrates, the nurses working in Poland were significantly older (45 vs. 41 years) and more experienced (10 vs. 8 years of experience) than those working in England. Regardless, the British sample was better educated (82% had a degree in nursing vs. 55% of the Polish sample) and had a higher number of male nurses (35% vs. 11%). The difference in education can be attributed to the fact that before joining the EU, nurses in Poland could be trained not only at universities but also in medical secondary schools. These schools provided five years of both general education and professional training, with graduation at the age of 19/20 years. All internationally trained nurses from the English sample obtained master’s degrees in their home countries. When coming to the UK, they were already registered with the Nursing and Midwifery Council (NMC); the UK was then in the European Union; that is, their diplomas were recognized by the NMC. The nurses trained in Britain graduated from nursing schools with bachelor’s degrees and were registered with the NMC.

### 2.2. Interview Questions

An 11-item structured interview-based survey was applied. It included six sociodemographic questions, three five-point Likert scale questions concerning nurses’ education and work satisfaction, as well as their promotion opportunities, two nominal-scale questions concerning participation in post-graduate training (yes/rarely/no), and promotion aspirations (yes/no). It was followed by two open-ended qualitative interview questions concerning the ups and downs of nurses’ education and work. The interview questionnaire developed for this study is available as an Appendix A. Each interview lasted for about 15 min. The qualitative answers were recorded and transcribed for further content analysis.

### 2.3. Inductive Coding

The qualitative narratives were read by two researchers (EW and MM) to localize the main recurring themes and topics that have to do with education (first open question) and work (second open question). Five thematic categories were thus inductively and consensually (meaning through discussion and agreement) identified regarding education—these were “theoretical knowledge”, “practical knowledge”, “finance”, “social dimension”, and “personal satisfaction”—and six further thematic categories were analogically identified regarding work—these were “professional development”, “state-of-the-art work environment”, “finance”, “social dimension”, “personal satisfaction”, and “teamwork”. The names of the categories were thus chosen by the researchers based on the content of the interview, which was certainly much broader and more nuanced than these names themselves. For example, the category of “theoretical knowledge” groups those contents that relate to the theoretical dimension of knowledge obtained during education, such as cognitive advantages, the level of lecturers, the difficulty of the study material, and the variation in the scope of teaching. The contents behind each category are presented in Table 2.

The categories “social dimension” and “personal satisfaction” appear in both questions; their content, however, is not identical. For example, personal satisfaction concerning education differs from one related to work, as represented by their respective contents in the second column.

### 2.4. Deductive Coding and Validation

Once the names of the categories and the content assigned to them were determined, the 258 respondents’ narratives were retrospectively coded for each of the categories as either a positive aspect (+), negative aspect (−), or non-occurrence (0). The categories could overlap; e.g., a respondent could emphasize that education has brought them great personal satisfaction (personal satisfaction +) and, at the same time, state that the knowledge they possessed does not translate into professional work (practical knowledge −). At this stage, therefore, the coding of statements was deductive in the sense that it referred to a ready and closed set of previously inductively constructed categories. Because most of the content behind the categories did not appear in the individual utterances due to the high number of categories, coding content as the “non-occurrence” of a category was the most common. To make the study results more accessible, the tables presenting significant differences only include data on each category’s positive or negative aspects. However, it should be noted that the “non-occurrence” only means that a particular respondent did not address a particular topic while answering the open-ended qualitative question. Therefore, not speaking of a particular topic, such as personal satisfaction, should not be treated as a lack of response but as the non-occurrence of content belonging to a given category. In this sense, it can be interpreted positively.

The content of the answers to the open-ended questions was coded, i.e., assigned to the available categories and valued (+, −, or 0) independently by two research team members (EW & MM), and the Kappa coefficient was calculated using GraphPad 9.0 software. In most cases, the strength of agreement was substantial and sometimes even almost perfect (see Table 3 for details). After assessing compliance, the final coding of the content was jointly determined.

### 2.5. Ethics Approval

All procedures contributing to this work comply with the ethical standards of the relevant national and institutional committees on human experimentation and the Helsinki Declaration of 1975, as revised in 2008. The Poznan University of Medical Sciences Bioethics Committee approved the survey as a non-experimental type (decision no. KB-341/24).

### 2.6. Statistical Analysis

Statistical analysis was performed using Statistica 13.0 (chi-square, Mann–Whitney, and Kruskal–Wallis tests).

## 3. Results

### 3.1. Education Satisfaction

Nurses of various nationalities working in England present significantly higher levels of education satisfaction as measured by the closed Likert scale question (55 vs. 3.88 on a five-point scale). The same can be observed when comparing the subsample of Polish nurses working in Poland and England (4.44 vs. 3.88 (see Table 4)). At the same time, qualitative interview answers concerning nurses’ education suggest that those working in England appreciate the theoretical knowledge acquired during undergraduate training more than those working in Poland. A total of 80% of the English sample and 78% of the Polish nurses in England mentioned in the interviews that their education provided them with high-quality theoretical knowledge needed in their work. In comparison, only 44% of nurses in Poland had the same opinion (see Table 4). Other aspects of nurses’ education mentioned in the open interview showed no differences in both analyses. The fact that the Polish nurses working in England tend to appreciate their education better can be somewhat surprising since they were originally educated in the same system as those who did not leave Poland.

### 3.2. Work Satisfaction

Surprisingly, the differences between work satisfaction levels among nurses in Poland and England and Polish nurses working in Poland and England (see Table 5) were not statistically significant when measured by the closed Likert question. However, the analysis of coded open-ended interview questions showed that nurses working in England are satisfied with their jobs and professional development more often than their Polish counterparts. They also felt financially rewarded and were satisfied with their state-of-the-art work environment. At the same time, the nurses in Poland more often expressed their financial dissatisfaction and problems with teamwork. Similar results were observed when we compared Polish nurses working in Poland and England. The main difference is that Polish nurses in England feel their profession is respected (16% of them mentioned that in the interviews). In contrast, no nurse in Poland expressed a similar opinion. Interestingly, there was no difference between Polish nurses in the two countries regarding personal satisfaction driven by work (see Table 5).

### 3.3. Promotion Opportunities and Aspirations

Regarding promotion opportunities for nurses working in Poland and England, the situation looks much better in Great Britain. In total, 79% of nurses working there (and 82% of Polish nurses in the UK) declare they would like to be promoted, while only 50% of Polish nurses expressed the same desire. No wonder more nurses participate in post-graduate training in England than in Poland (see Table 6). This can be attributed to the differences in the nursing systems between the countries. The British band system creates clear professional development paths, while in Poland, nurses can only work as regular unit nurses or choose from the positions of the unit head nurse, hospital head nurse, or the hospital’s epidemiological nurse, these positions being very limited within the system. It is therefore not surprising that their willingness to upgrade their qualifications is not as strong as in England.

## 4. Discussion

### 4.1. Education

The survey showed that nurses working in England, regardless of their nationality, are significantly more satisfied with the education they received than nurses working in Poland. Since Polish nurses in England and Poland were educated in the same Polish education system, one may wonder if this difference is not related to the improvement of the economic and living conditions of the nurses who decided to migrate to the UK, which, in a way, also benefited them (thanks to the education they received) in many spheres of professional and personal life. Polish nurses working in Poland see few advantages to their education compared to other medical professional groups. Other studies show that education and qualification improvement provide nurses neither motivation, as promotion is unrealistic, nor concrete financial gratification, and most often only personal satisfaction [17]. Usually, only the financial aspect motivates nurses to undertake further education and training [18]. It should be noted that in hospitals in Poland, before the salary scale was set, financial allowances were not always provided to those with higher qualifications, demotivating those eager to continue their education.

Studies evaluating the level of satisfaction with the education of nurses working in various departments show that nurses emphasized that education provided them a very good theoretical preparation. Regardless, from the practical point of view, only the work solidified their choice of profession and prepared them to practice it fully professionally [17,18]. These results were also confirmed in the present study. The surveyed Polish nurses and nurses working in England were more likely to mark the advantages of the theoretical aspects of education than the practical ones. It is, therefore, worth changing the curriculum to help future workers and not to increase the number of practical classes in nursing faculties. Even though English nurses are satisfied and operate well in both their education and work in the profession, it is recommended that the quality of education systems be periodically inspected and evaluated, both from a practical and theoretical perspective [19].

### 4.2. Work

Regarding job satisfaction, no significant differences were found between nurses working in Poland and England or between Poles working in Poland and England. It is essential to consider why all respondents who answered the closed question about their work declared satisfaction. We can easily understand this by evaluating the workplace and all the related quality aspects of English hospitals. At the same time, it may be a surprise that Polish nurses, despite the voices of dissatisfaction in the media, do not differ from the other surveyed nurses in terms of satisfaction levels. Perhaps this relates to getting used to Polish hospitals’ current state of affairs. However, one should inquire whether such an attitude is good for employees and employers. On the one hand, accepting the state of affairs helps them survive in specific conditions that are not entirely favourable. On the other hand, coming to terms with the situation will not motivate them to change it or take up post-graduate training. All the negative aspects of work, such as overload, low satisfaction, failure to find one’s place in the team, or inadequate financial compensation, can lead to burnout or depression. This aspect is often noted and addressed in research [20,21,22]. Analyzing the group studied in this paper from this angle, an additional risk factor is the particular and demanding work environment (the intensive care units). A possible consequence is professional burnout, which can even lead to leaving the profession. Research on the level of job satisfaction of nurses (related to work organization system and conditions, socio-behavioral factors, and a good atmosphere at work [23]) is therefore vital. It may enable the early detection of the potential risks associated with the deterioration of nurses’ mental health. In this context, it is also important to mention the problem of understaffing in hospitals in Poland and the UK [24,25,26].

### 4.3. Promotion

It is worth noting that there has been very little research on evaluating promotion in nursing. This is due to the very limited opportunities for such promotion in Poland. Polish ward nurses, regardless of their level of education, specialist training, and work experience, perform exactly the same tasks. Becoming a Ward Head Nurse or a Hospital Head Nurse is the only option for formal promotion in Poland. Such a nurse is responsible for managing the nursing staff; however, there can be only one per ward or hospital. In contrast, the UK has a clearly defined and more diverse career development and advancement path (e.g., the NHS Nursing Bands), with many specialties to choose from. It was noted that nurses working in the UK are significantly more likely to receive post-graduate training than employees in Poland. A difference was also observed in the frequency of training attendance between Poles working in England and those working in Poland. As it turned out, people of Polish origin working in England were more likely to improve their skills through training than in Poland. Nevertheless, it should be noted that more than half of nurses in Poland attended training courses. Speaking of the whole continuing education process, a very good initiative in the UK, which is increasingly emerging in Poland, is the analysis of training systems to fix and improve them. Researchers have described this system as CPE (continuous professional education) [27,28,29]. While this system is already working well in the UK, it is unfortunately only in its early stages in Poland.

### 4.4. Limitations of the Study

The main limitation of this study is that the convenience sampling method affects the generalizability of results, as does the fact that the data were collected regionally—in Poznan and Puszczykowo, located in the Great Poland region, and the hospitals in Guilford and Papworth Everard, situated in two distinct regions of England. Due to time constraints, the answers to open questions were also relatively short for qualitative studies, which was nevertheless balanced by a large sample size for qualitative studies. It would be desirable to compare the findings with an even larger group and one including subjects from more diverse regions.

## 5. Conclusions

The professional situation of Polish nurses working in Poland is not optimistic. Regarding satisfaction with education, nurses working in England have an advantage, regardless of their nationality and where they obtained their diploma. An open-ended question on the advantages and disadvantages of the job revealed significant differences in the perception of the profession in favor of the UK. There is a multitude of areas in Polish nursing that require reform. Nurses working in England are more likely to perceive an opportunity and be willing to advance professionally. They are also more likely to participate in continuing education, to which they have easier access, thanks to the subsidies they receive from British employers. Unfortunately, the path to promotion among nurses in Poland is still not generally available. This fact undeniably influences nurses’ lack of effort in Poland to obtain a promotion, as they do not see its benefits. Becoming a ward/hospital head nurse and managing the work of your former peers requires a specific set of skills and is often perceived as a nuisance by many nurses.

This research indicates that the key variable critical to the level of satisfaction and opportunities for professional development is neither nationality nor country of education but the work system. The latter involves the components such as equipment and infrastructure, work atmosphere, salary, employer support, and the feeling of being part of a team. It is, therefore, advisable to strive for continuous change and improvement of the working system of nurses in Poland and model it on the British system. The latter transparently differentiates the professional tasks and responsibilities of nurses and their remuneration according to qualifications and experience. Creating a clear and fair career planning and development system with an increased diversity of professional development paths that are researchable step by step is thus a desirable direction for the Polish nursing system. Such a system would likely encourage more nurses in Poland to participate in postgraduate training, raise their job satisfaction, and reduce the migration of nursing professionals.

## Figures and Tables

**Table 1:** Sample characteristics (*n* = 258).

	Poland	England	Total
**Sample**	*n* = 72 (28%)	*n* = 186 (72%)	*n* = 258
Age *	$\bar{X}$ = 45 min = 28 max = 61 Q1 = 36 Q2 = 45 Q3 = 54 SD = 9.48	$\bar{X}$ = 41, min = 27 max = 62 Q1 = 32 Q2 = 40 Q3 = 48 SD = 9.99	$\bar{X}$ = 42 min = 27 max = 62 Q1 = 33 Q2 = 41 Q3 = 50 SD = 9.98
Sex **	Female = 64 (89%) Male = 8 (11%)	Female = 120 (65%) Male = 66 (35%)	Female = 184 (71.3%) Male = 74 (28.7%)
Work experience (in years) ***	$\bar{X}$ = 10 min = 2 max = 23 Q1 = 7 Q2 = 10 Q3 = 12 SD = 5.25	$\bar{X}$ = 8 min = 1 max = 23 Q1 = 4, Q2 = 7, Q3 = 11, SD = 4.4	$\bar{X}$ = 8.5 min = 1 max = 23 Q1 = 5 Q2 = 8 Q3 = 11 SD = 4.76
Education ****	Medical secondary school 32 (45%) Bachelor’s Degree in Nursing 24 (33%) Master’s Degree in Nursing 16 (22%)	Medical secondary school 33 (18%) Bachelor’s Degree in Nursing 120 (64%) Master’s Degree in Nursing 33 (18%)	Medical secondary school 65 (25.2%) Bachelor’s Degree in Nursing 144 (55.8%) Master’s Degree in Nursing 49 (19%)
**Nurses’ nationality**	Polish—72 (100%)	British—61 (32.8%) Polish—50 (26.9%) Portuguese—30 (16.12%) Spanish—25 (13.43%) Italian—20 (10.75%)	Polish—122 (47.28%) British—61 (23.63%) Portuguese—30 (11.63%) Spanish—25 (9.69%) Italian—20 (7.75%)

$\bar{X}$ = mean, Q1—bottom quartile, Q2—median, Q3—upper quartile, min—minimum, max—maximum, SD—standard derivation. * Mann–Whitney’s *U* = 1382.5; *p* = 0.029946, ** Chi^2^(1)) = 8.03; *p* = 0.00459. *** Mann–Whitney’s *U* = 1261.00 *p* < 0.00001, **** Chi^2^(2) = 24.68; *p* < 0.00001.

**Table 2:** Categories concerning the open questions on education and work.

**EDUCATION**	
**Category name**	**Category refers to**
Theoretical knowledge	Cognitive benefits of education itself or lack thereof High or low level of education Easy or difficult material to assimilate Good or poor lecturers Diversity of the scope of education or lack of it
Practical knowledge	Knowledge with or without translation into the job Content of education matching or not matching working conditions in a given country Availability or lack of professional practice Usefulness or uselessness of the diploma Education as a waste of time (the need for further education) or as time well spent
Finance	Positive or negative translation of education into future earnings
Social dimension	Positive or negative relationships and social contacts Prestige of education or lack thereof Benefits in the form of opportunities to work in other countries (language skills) or lack thereof
Personal satisfaction	Good memories or lack thereof Pride in education or lack thereof Reaching vocation or lack thereof
**WORK**	
**Category name**	**Category refers to**
Professional development	Additional training funded by the employer or lack thereof Clear opportunities for promotion and career development paths or lack thereof Positive or negative translation of knowledge into professional work Support from the employer or lack thereof
State-of-the-art work environment	Clear and transparent or unclear and non-transparent procedures of conduct Clear rules of work, good organization or lack thereof (chaos, disorganization) Modern equipment, technologies, medicines or lack thereof Sense of working in a modern or under-developed profession Adequate number of personnel or their shortage
Teamwork	Good or bad atmosphere of cooperation in the team Absence or presence of communication and language problems in the team Racist conflicts or lack of them Sense of being part of the team or sense of alienation in the environment
Finance	Good or bad salaries (the need to work several jobs) Sense of social security or lack thereof Training subsidized by the employer or lack thereof
Social dimension	Prestige or lack of respect toward the nursing profession Sense of being appreciated by patients and their families or lack thereof (resentment on their part)
Personal satisfaction	Work as a dream come true or reconciliation with work as fate (not the height of dreams) Proximity and time for family or separation from family and feeling lonely Job satisfaction, lack of overload, or work overload (stress, fatigue, emotional problems) Work as a vocation (mission accomplishment) or necessity satisfaction with helping and caring for patients or lack thereof

**Table 3:** Inter-rater reliability of coding the open questions.

**Categories regarding education**	**Value of k**	**Strength of agreement**
Theoretical knowledge	0.555	Moderate
Practical knowledge	0.538	Moderate
Finance	0.839	Almost perfect
Social dimension	0.775	Substantial
Personal satisfaction	0.667	Substantial
**Categories regarding work**	**Value of k**	**Strength of agreement**
Professional development	0.612	Substantial
State-of-the-art work environment	0.807	Almost perfect
Teamwork	0.748	Substantial
Finance	0.650	Substantial
Social dimension	0.765	Substantial
Personal satisfaction	0.845	Almost perfect

**Table 4:** Education satisfaction: Poland vs. England (*n* = 258) and Poland vs. Polish nurses in England (*n* = 122).

	Poland	England	Polish Nurses in England	Poland vs. England	Poland vs. Polish Nurses in England
Education satisfaction (on a 1 to 5 scale)	3.88	4.55	4.44	Mann–Whitney’s *U* = 1287.5; *p* = 0.033926	Mann–Whitney’s *U* = 1259.0; *p* = 0.016938
Education satisfaction (open-interview category) *Theoretical knowledge*	44%	80%	78%	Chi^2^(2) = 25.57; *p* < 0.00001	Chi^2^(2) = 15.71; *p* = 0.00039

There are no differences in other categories regarding education, that is, *practical knowledge*, *finance, social dimension*, and *personal satisfaction from education.*

**Table 5:** Work satisfaction: Poland vs. England (*n* = 258) and Poland vs. Polish nurses in England (*n* = 122).

	Poland	England	Polish Nurses in England	Poland vs. England	Poland vs. Polish Nurses in England
Work satisfaction (on a 1 to 5 scale)	3.18	3.69	3.82	*p* > 0.05	*p* > 0.05
Work Satisfaction (open-interview categories)					
*Professional development*	1%	15%	26%	Chi^2^(2) = 22.96; *p* < 0.00001	Chi^2^(2) = 19,76; *p* = 0.00005
*Personal satisfaction*	0%	11%	3%	Chi^2^(2) = 24.05; *p* < 0.00001	*p* > 0.05
*Problems with Teamwork*	14%	5%	0%	Chi^2^(2) = 15.65; *p* = 0.0004	Chi^2^(2) = 10,14; *p* = 0.00627
*State-of-the-art work environment*	0%	13%	16%	Chi^2^(2) = 15.25; *p* = 0.00049	Chi^2^(2) = 17,48; *p* = 0.00016
*Financially satisfied*	0%	24%	34%	Chi^2^(2) = 56.34; *p* < 0.00001	Chi^2^(2) = 41,41; *p* < 0.00001,
*Financially dissatisfied*	39%	3%	2%	Chi^2^(2) = 56.34; *p* < 0.00001	Chi^2^(2) = 41,41; *p* < 0.00001,
*Social dimension/Prestige*	0%	8%	16%	*p* > 0.05	Chi^2^(2) = 17,48; *p* = 0.00016

**Table 6:** Promotion opportunities and aspirations: Poland vs. England (*n* = 258) and Poland vs. Polish nurses in England (*n* = 122).

	Poland	England	Polish Nurses in England	Poland vs. England	Poland vs. Polish Nurses in England
Promotion opportunities (on a 1 to 5 scale)	2.63	4.42	4.68	Mann–Whitney’s *U* = 1349.5; *p* = 0.032527	Mann–Whitney’s *U* = 1621.0; *p* = 0.03749
Promotion aspirations (yes/no)	50%	79%	86%	Chi^2^(1) = 39.34; *p* < 0.00001	Chi^2^(1) = 12.97, *p* = 0.00031
Participation in post-graduation training					
Yes	62%	86%	100%	Chi^2^(2) = 72.00; *p* < 0.00001	Chi^2^(2) = 24.07, *p* = 0.00001
Rarely	25%	9%	0%		
No	13%	5%	0%		

## Data Availability

Raw data upon which this study is based are available upon request.

## Public Involvement Statement

No public involvement in any aspect of this research.

## Appendix A

### Appendix A.1. Interview Questionnaire Part 1: Quantitative

1. Sex
2. Age
3. Country of work
  - Poland
  - Great Britain
4. Country of origin
  - Great Britain
  - Italy
  - Spain
  - Poland
  - Portugal
5. Level of education
  - Medical secondary school
  - Bachelor’s degree in Nursing
  - Master’s degree in Nursing
  - PhD
6. Work experience (in years)
7. Are you satisfied with your education?
  - Very satisfied
  - Satisfied
  - Neither satisfied nor dissatisfied
  - Dissatisfied
  - Very dissatisfied
8. Do you take part in post-graduate training?
  - Yes
  - Rarely
  - No
9. Are there promotion opportunities in your work?
  - Most of the time
  - Some of the time
  - I do not know
  - Seldom
  - Never
10. Are you interested in professional promotion?
  - Yes
  - No
11. Are you satisfied with your work?
  - Very satisfied
  - Satisfied
  - Neither satisfied nor dissatisfied
  - Dissatisfied
  - Very dissatisfied

### Appendix A.2. Interview Questionnaire Part 2: Qualitative (Open Verbal Interview Questions)

1. What are the ups and downs of your education?
2. What are the ups and downs of your work as an intensive care unit nurse?
